# Giant myxofibrosarcoma of neck: A case report and review of the literature

**DOI:** 10.1097/MD.0000000000042150

**Published:** 2025-04-18

**Authors:** Linkun Zhong, Ying Zhang, Hongjian Kang, Zhaohua Wang

**Affiliations:** a Department of General Surgery, Zhongshan City People’s Hospital, Zhongshan, Guangdong Province, China; b Department of Otorhinolaryngology & Head and Neck Surgery, Dalian Municipal Friendship Hospital, Dalian, Liaoning Province, China; c Department of Medical Record Room, Zhongshan City People’s Hospital, Zhongshan, Guangdong Province, China.

**Keywords:** case report, myxofibrosarcoma, neck, soft tissue sarcoma

## Abstract

**Rationale:**

Myxofibrosarcoma (MFS) is a subtype of soft tissue sarcoma that commonly occurs in the extremities but is rare in the neck. It is characterized by a high risk of local recurrence due to its specific infiltrative growth pattern.

**Patient concerns:**

A 56-year-old male presented with a large mass in the right neck with disability of the right upper limb.

**Diagnoses:**

Magnetic resonance imaging revealed a 12.48 × 9.01 × 14.16 cm mass with a “tail sign.” Resection with wide margins was performed to remove the tumor and the invaded surrounding tissues. Histopathological examination confirmed the diagnosis of high-grade MFS.

**Interventions:**

The patient developed local recurrence 24 months after surgery and died of the disease 26 months after the recurrence.

**Outcomes:**

This case presents the largest MFS located in the neck reported to date. The treatment of giant MFS in the neck is challenging due to the proximity of vital structures including vessels, nerves, and other tissues. The invasive pattern of MFS facilitates extensive spread into surrounding tissues, complicating complete surgical removal. And insufficient margins frequently result in disease recurrence.

**Lessons:**

This case highlights the significance of wide surgical margins in instances of extensive neck infiltration by MFS. However, further studies are required to confirm the specific margin values, and the advancement of effective adjuvant therapy is essential for improving patient survival.

## 1. Introduction

Myxofibrosarcoma (MFS), also known as malignant fibrous histiocytoma, was officially first reported in 1977.^[1]^ MFS is a distinctive subtype of soft tissue sarcoma that frequently occurred in connective tissue, particularly fibrous and fatty tissue of elderly adults. It is characterized by a mixture of mucus and fibrous components. According to the World Health Organization 2020 classification of soft tissue tumors, MFS is a fibroblastic/myofibroblastic tumor representing approximately 5% of soft tissue sarcoma.^[2]^ MFS is primarily present in the extremities and trunk, while it is extremely rare in the neck.^[3,4]^ We present a case of giant MFS of the neck in this report. To the best of our knowledge, this is the largest MFS located in the neck region.

## 2. Case report

**Figure 1.**
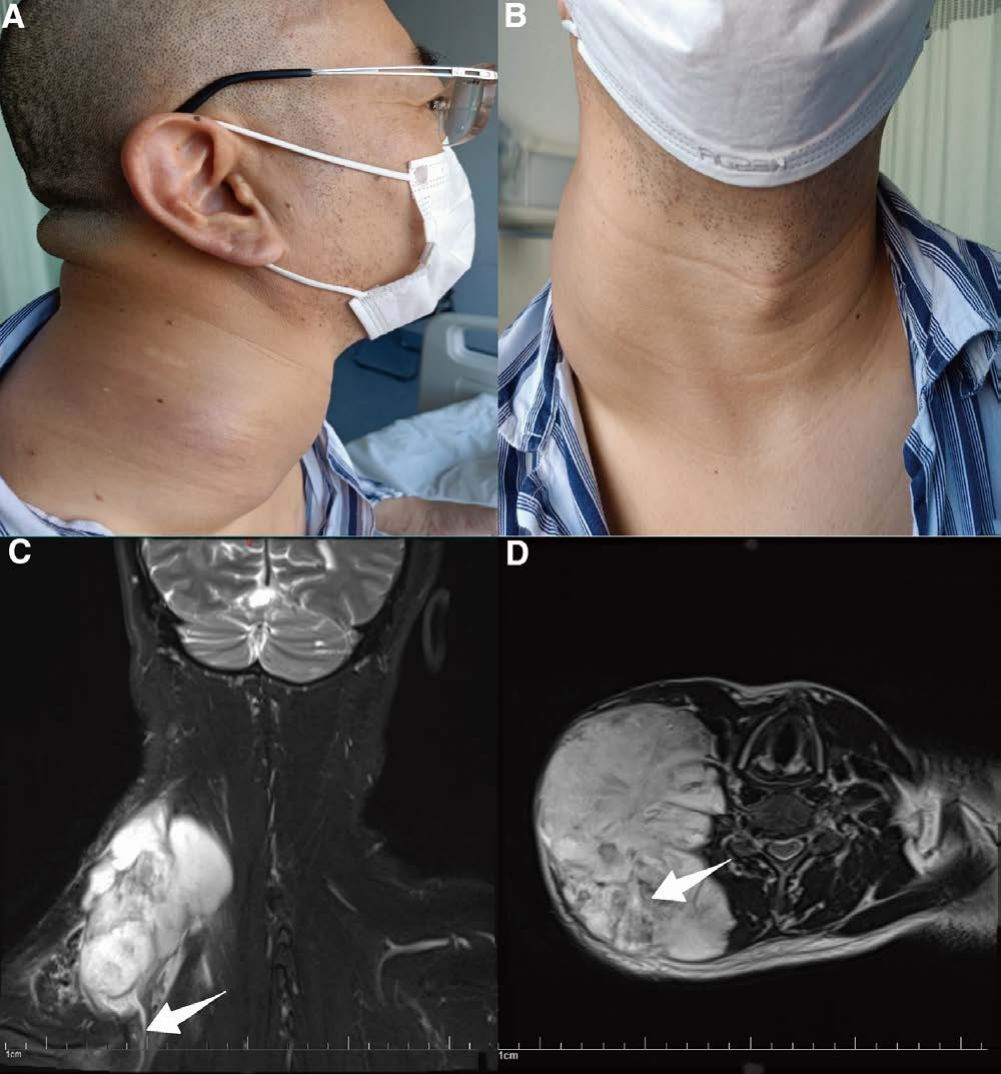
Images of clinical examination. (A, B) Clinical images showed a giant mass in the left neck. (C) MRI showed low signal intensity on T1WI and “tail sign” (arrow). (D) MRI showed high signal intensity on T2WI with separate pattern inside the tumor (arrow).

A 56-year-old Chinese man presented with a giant painless mass in his right neck, which gradually increased in size over 18 months (Fig. 1A, B). The patient observed the rapid growth and limitation of the right upper extremity in the past 2 months. He visited a local hospital for a needle core biopsy. The pathological examination indicated that the tumor contained active spindle cells and muscle fibers. There was no history of trauma, radiation exposure, infection, or surgery. The patient was admitted to the hospital for surgery treatment on April 23rd, 2020. The preoperative magnetic resonance imaging (MRI) revealed a 12.48 × 9.01 × 14.16 cm mass from the atlas to the first thoracic vertebrae level. The tumor exhibited a low signal intensity on T1WI and a high signal intensity on T2WI with a “tail sign” and separate pattern, respectively (Fig. 1C, D). The electromyography indicated an incomplete injury of the right brachial plexus and accessory nerve (Table 1).

**Table 1:** Electromyographic examination report sensory nerve conduction studies.

Nerve	Lat (ms)	Amp (uv)	CV (m/s)
Lateral antebrachial cutaneous nerve (sensation left)			
Elbow–elbow blow	1.06	69.8	75.5
Lateral antebrachial cutaneous nerve (sensation right)			
Elbow–elbow blow	1.02	32.5	68.6
EMG MUP data			
Muscle (right)	Resting potential	MUP	IP (mv)
Deltoid	P++++ f++++	(+)	1.6
Infraspinatus	P+++ f+	(+)	(‐)
Trapezius	P+++++ f+++++	(+)	(‐)
Biceps brachii	P+++++ f+++++	(+)	2.6
Brachioradialis	P++++ f++++	(+)	3.0

**Figure 2.**
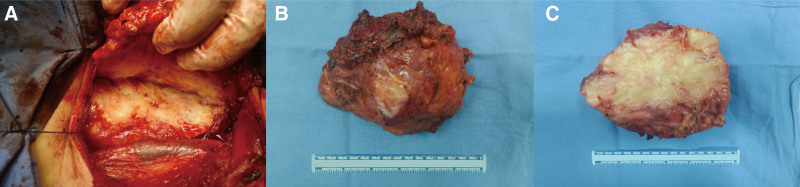
Images of tumor during operation. (A) Intraoperative images of tumor. (B, C) Images of tumor after resection.

**Figure 3.**
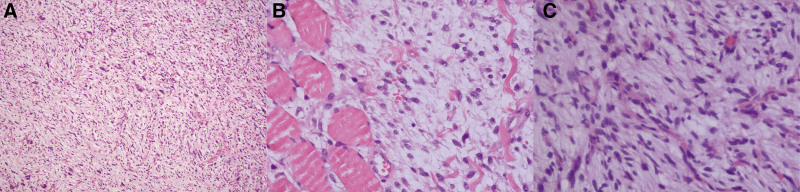
Histological examination of tumor. (A) Photomicrograph showed spindle cells, myxoid matrix and cell aggregation (H&E stain, 100×). (B) Photomicrograph showed tumor cells among muscle fibers and arcuate vessels. (H&E stain, 400×). (C) Photomicrograph showed pleomorphism of tumor cells. (H&E stain, 400×).

**Figure 4.**
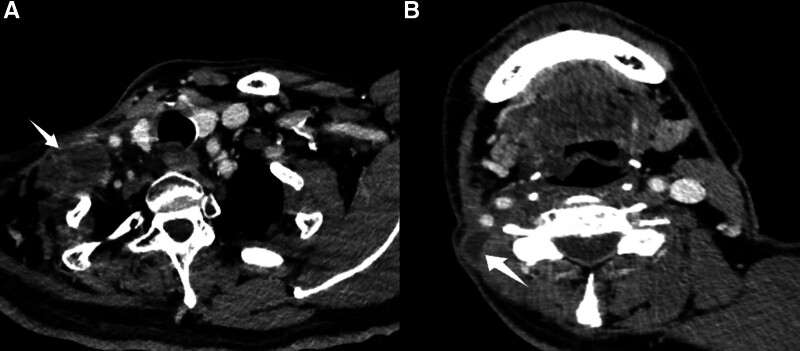
Images of MFS recurrence. (A, B) The enhanced CT showed local recurrence of tumor in the right neck (arrow).

We removed the tumor and its invaded adjoining tissues, including the sternocleidomastoid, omohyoid, trapezius, scapulae, external jugular vein, cervical plexus, lymph nodes, accessory nerve, and brachial plexus. The resected tumor was macroscopically soft and yellow-white, and its surface was uniformly gelatinous and myxoid (Fig. 2). Additionally, we obtained a negative margin 1 cm away from the tumor tissue. Histological examination revealed that the lobulated tumor and brachial plexus were composed of spindle-shaped cells with a predominantly myxoid background. Arcuate vessels and muscle fibers were found among the tumor cells. The tumor necrosis cells had crowded nuclei with hyperchromatism and pleomorphism at high magnification (Fig. 3). The immunohistochemical staining exhibited CD34, patchy positivity for vimentin, and Ki67 (+40%). SMA, desmin, cytokeratin (AE1/AE3) and S-100 were negative. Based on the above results, the diagnosis was confirmed to be high-grade MFS (grade 3). Gene sequencing of the tumor identified the mutation of ATRX, ERCC4, MYB, NLRCS, NTRK1, TP53, TRAF7, RB1, and MSH. The patient underwent radiation therapy and chemotherapy, followed by the development of local recurrence in the right neck 23 months after surgery (Fig. 4). Unfortunately, the patient did not survive the disease 26 months after the recurrence.

## 3. Discussion

As a soft tissue sarcoma, MFS occurs primarily in the subcutaneous or dermis tissue and occasionally in subfascial or muscle tissue.^[5]^ The incidence of MFS in the neck region is extremely low, with only 8 cases reported in the relevant literature. The clinical characteristics of these cases are summarized in Table 2.^[6–13]^

**Table 2:** Cases of myxofibrosarcoma in the neck.

Sex	Age	Location	Treatment	Results	Reference
Male	69	Hypopharynx	Surgery	Alive after 16 months	^[6]^
Male	55	Neck	Surgery	Alive after 27 months	^[7]^
Male	79	Vocal fold	Surgery	Unknown	^[8]^
Male	42	Sinus piriformis	Surgery	Alive after 46 months	^[9]^
Female	74	Thyroid gland	Surgery/chemotherapy	Unknown	^[10]^
Male	64	Neck and shoulder	Surgery	Local recurrence after 83 months	^[11]^
Male	48	Neck and chest	Surgery	Unknown	^[12]^
Male	67	Neck	Surgery	Alive after 24 months	^[13]^

Surgery is the primary therapeutic approach for managing MFS. However, giant MFS is difficult to manipulate. The primary objective of surgery is to delineate the tumor margin and completely remove it. Sanfilippo et al suggested that size and margin were positively correlated, indicating that patients with positive margins often had larger tumors.^[14]^ And another study indicated that intermediate and high-grade MFS median size was 7.5 cm. And the survival rate decreased with the increasing tumor diameter.^[15]^ The patient’s delay in seeking medical attention as a result of the global COVID-19 pandemic resulted in a larger tumor size upon their arrival at our department. Electromyography revealed signs of incomplete nerve injury, suggesting a potential invasion of nerve tissue by the tumor. The boundaries between the tumor and surrounding normal tissue were indistinct during the surgery. This highlighted that an invasive growth pattern typically characterized MFS and extended into adjacent connective tissue, complicating the assessment of tumor size.^[16]^

It can be difficult to accurately confirm the margin of the tumor during surgery for giant MFS. Preoperative MRI is crucial for the surgeon to easily identify tumor margins. MFS typically exhibits a “tail sign” on MRI, serving as a specific indicator for diagnosing MFS and influencing the likelihood of local recurrence.^[17–19]^ We did not find the exact “tail sign” tumor tissues during the operation. Therefore, we performed an extended tumor resection to obtain a negative margin. Previous studies indicated that surgical margin was the predictive factor of local recurrence.^[20,21]^ Odei et al reported that 67% and 56% of local recurrence patients had high grade and large tumor diameters (>5 cm).^[22]^ Patients with positive margins were more likely to have local recurrence than those with negative ones. Furthermore, patients with resection margins of > 1 mm had longer local recurrence-free survival and overall survival.^[23]^ However, Tomas et al reported that a 2 mm margin was not enough to affect the recurrence rate. Clear margin had a positive effect on recurrence-free survival; however, it did not significantly affect the overall survival.^[24]^ Besides, Sambri et al suggested that surgical margin did not affect prognosis in grade 3 MFS.^[25]^ The above conclusions are based on the MFS in extremities and trunk. However, there is limited evidence to support whether a positive margin is associated with the prognosis in the neck. In this case, even with a 1 cm negative margin obtained near the tumor, a risk of missing invaded tissue existed due to the biological characteristics of MFS, resulting in disease recurrence. Consequently, further studies are required to confirm the specific margin value for giant MFS.

Negative resection margins are crucial to ensure the complete resection of tumors as observed macroscopically. However, due to MFS’s unique infiltrative growth patterns, positive resection margins can be easily overlooked. A previous study indicated that MFS with infiltrative growth patterns frequently exhibited an increased presence of mucinous areas and a higher local recurrence rate. Compared to normal blood vessels, MFS was characterized by more sickle-shaped blood vessels and fewer αSMA-positive cells. The increase in sickle-shaped blood vessels complicated the achievement of negative resection margins and was significantly associated with distant metastasis, overall survival rates, and poor prognosis.^[26]^ Determining tumor location and extent predominantly depends on conventional imaging examinations, including computed tomography and MRI before surgery. Among these, MRI is the most sensitive and accurate tool for determining the resection margin. A broader resection margin is required during surgery to encompass the entire tissue associated with the “tail sign.”^[27]^ In the context of a giant MFS, the presence of a vascular pedicle adjacent to the tumor, coupled with the persistence of the “tail sign” as evidenced by MRI posttreatment, suggests an increased disease recurrence rate and a significant decrease in patient survival rates.^[28]^ Recently, a novel technology utilizing near-infrared fluorescence imaging has been developed. This innovative approach employs specific tumor biomarkers to identify tumors during surgical procedures. Notably, TEM1, VEGFR-1, EGFR, VEGFR-2, IGF-1R, PDGFRα, and CD40 are highly expressed in the tumor cells of soft tissue sarcoma. The conjugation of these antibodies with fluorescent dyes serves as specific tracers in image-guided surgery, thereby increasing the success rate of tumor resection.^[29,30]^

Comprehensive genomic analysis offers a profound understanding of the molecular drivers of sarcoma. In a large-sample study on sarcoma, gene sequencing could refine or reassign of 10.5% of diagnoses. Based on these results, treatment strategies were changed for nearly one-third of patients. These changes included kinase gene rearrangements and a tumor mutation burden ≥ 10 mut/Mb, low frequencies of microsatellite instability, and a high degree of genome-wide loss of heterozygosity.^[31]^ Adults with soft tissue sarcomas tend to have low somatic mutation burdens and with a few genes (TP53, ATRX, and RB1) highly mutated across different sarcoma types.^[32]^ A recent gene sequencing study suggested that common soft tissue sarcomas, MFS, and undifferentiated pleomorphic sarcoma, exhibited a lower tumor mutation burden and significant overlap in their genomic and transcriptomic characteristics. Notably, the incidence of whole-genome doubling in MFS was lower than that in undifferentiated pleomorphic sarcoma, indicating a relatively reduced genomic complexity in MFS.^[33]^ Additionally, other driver genes have been identified, including ATRX, JAK1, NF1, NTRK1, and BRAF fusion genes.^[34]^ Because of mutations in these genes, MFS is refractory to cytotoxic chemotherapy and radiotherapy. In advanced and metastatic diseases, anthracycline-based chemotherapy regimens, including daunorubicin, doxorubicin, and epirubicin are one of the primary treatment strategies. Identifying potential biomarkers for chemotherapy response can facilitate the development of personalized treatment strategies for each patient.^[35]^ Vanni et al identified AKR1C2, AKR1C3, BMP7, and SGC as significant markers of aggressive MFS. AKR1C3, a crucial carbonyl reductase, exhibited up-regulation in MFS, which was a key factor contributing to the up-regulation and drug resistance during adriamycin metabolism.^[36]^ This suggest that developing and applying carbonyl reductase inhibitors can provide therapeutic benefits for patients. Furthermore, additional research is required to identify markers specific to MFS to facilitate the implementation of effective targeted therapies.

The primary challenge in managing the recurrence of MFS is the absence of effective systemic treatments. MFS frequently exhibits partial drug resistance or variable efficacy in response to standardized chemotherapy. The distinct biological behaviors and drug sensitivities of tumor cells isolated from individual patients contribute to significant heterogeneity. Moreover, there is an urgent need for additional biological targets to enhance treatment efficacy. The availability of appropriate in vitro cell lines is essential due to the frequent observation of intratumoral and intertumoral cytogenetic heterogeneity and clonal evolution in MFS. However, only a limited number of patient-derived MFS cell lines have been documented. Guerrieri et al developed a novel patient-derived MFS cell line (MF-R3), which exhibited the invasive characteristics of MFS and demonstrated chemosensitivity to anthracycline drugs under both two-dimensional and three-dimensional culture conditions.^[37]^ Consequently, establishing additional MFS cell lines will enhance our understanding of the pathogenesis and drug response associated with MFS. The most prevalent copy number alterations in MFS involve RB1 and TP53, which encode the Rb and p53 tumor suppressor proteins, respectively. MFS without Rb and p53 depends on the Skp2 oncogene for survival. However, MFS can be suppressed by the inhibitor Pevonedistat, which can target Skp2.^[38]^ On the other hand, TRIO and RICTOR amplification can be observed at different levels in MFS.^[39]^ Okada et al identified that ITGA10 gene was significantly associated with disease-specific mortality and distant metastasis in high-grade MFS. ITGA10 transmitted specific signals through TRIO and RICTOR, activating RAC/PAK and AKT/mTOR pathways to promote tumor cell survival. The combination of RAC inhibitors and mTOR inhibitors significantly inhibited MFS growth.^[40]^ Moreover, from an immunomics perspective, sarcomas with complex karyotypes, including MFS, exhibit a tumor microenvironment characterized by a high degree of immune infiltration, which increases the likelihood of response to immunotherapy.^[41]^ The expression levels of immune microenvironment markers, including B7-H3, TGF-β1, and TIM-3, as well as the immune infiltration score, are elevated in MFS. Additionally, the immune infiltration of NK cells is correlated with improved disease-specific survival.^[32]^ Higher levels of PD-L1, PD-1, and tumor-infiltrating lymphocytes are observed in MFS compared to other soft tissue sarcomas. Moreover, regulatory T cells are associated with an increased risk of local recurrence; however, not with margin positivity.^[42]^ Consequently, several studies have demonstrated that PD-1 inhibitors, either alone or in combination with other agents, can effectively regulate the MFS progression.^[43,44]^

## 4. Conclusion

In summary, the key to managing MFS in the neck is the completely removal of the tumor and invaded tissues. However, extensive tissue invasion caused by giant tumors and insufficient surgical margins remain significant challenges that need to be addressed. Additionally, further research is required to explore specific chemotherapy and targeted therapies, aiming to develop treatment strategies that significantly reduce recurrence rates and enhance long-term clinical outcomes for patients.

## Author contributions

**Conceptualization:** Linkun Zhong, Zhaohua Wang.

**Data curation:** Ying Zhang, Hongjian Kang.

**Funding acquisition:** Linkun Zhong.

**Investigation:** Zhaohua Wang.

**Methodology:** Zhaohua Wang.

**Project administration:** Zhaohua Wang.

**Validation:** Zhaohua Wang.

**Visualization:** Hongjian Kang.

**Writing – original draft:** Linkun Zhong, Zhaohua Wang.

**Writing – review & editing:** Zhaohua Wang.
